# Supplementary material for: Biochemical and Thermodynamic Studies on a Novel Thermotolerant GH10 Xylanase from Bacillus safensis
Source: Biomolecules. 2022 Jun 6;12(6):790. doi: 10.3390/biom12060790 (PMC9221164; doi:10.3390/biom12060790)
Supplement: Supplementary file 1 [file biomolecules-12-00790-s001.zip › supplementary Figure S1.pdf]

## Supplementary Figure S1

*B. safensis* ATHUBA63 16S rDNA [Accession Number: ON430671]

GACGAACGCTGGCGGCGTGCCTAATACATGCAAGTCGAGCGGACAGAAGGGAGCTTGCTCCCGGATGTTAGCGGC
GGACGGGTGAGTAACACGTGGGTAACCTGCCTGTAAGACTGGGATAACTCCGGGAAACCGGAGCTAATACCGGAT
AGTTCCTTGAACCGCATGGTTCAAGGATGAAAGACGGTTTCGGCTGTCACTTACAGATGGACCCGCGGCGCATTAG
CTAGTTGGTGGGGTAATGGCTCACCAAGGCGACGATGCGTAGCCGACCTGAGAGGGTGATCGGCCACACTGGGAC
TGAGACACGGCCCAGACTCCTACGGGAGGCAGCAGTAGGGAATCTTCCGCAATGGACGAAAGTCTGACGGAGCA
ACGCCGCGTGAGTGATGAAGGTTTTCGGATCGTAAAGCTCTGTTGTTAGGGAAGAACAAGTGCGAGAGTAACTGC
TCGCACCTTGACGGTACCTAACCAGAAAGCCACGGCTAACTACGTGCCAGCAGCCGCGGTAATACGTAGGTGGCA
AGCGTTGTCCGGAATTATTGGGCGTAAAGGGCTCGCAGGCGGTTTCTTAAGTCTGATGTGAAAGCCCCCGGCTCAA
CCGGGGAGGGTCATTGGAAACTGGGAAACTTGAGTGCAGAAGAGGAGAGTGGAATTCCACGTGTAGCGGTGAAA
TGCGTAGAGATGTGGAGGAACACCAGTGGCGAAGGCGACTCTCTGGTCTGTAACTGACGCTGAGGAGCGAAAGCG
TGGGGAGCGAACAGGATTAGATACCCTGGTAGTCCACGCCGTAAACGATGAGTGCTAAGTGTTAGGGGGTTTCCG
CCCCTTAGTGCTGCAGCTAACGCATTAAGCACTCCGCCTGGGGAGTACGGTCGCAAGACTGAAACTCAAAGGAAT
TGACGGGGGCCCGCACAAGCGGTGGAGCATGTGGTTTAATTCGAAGCAACGCGAAGAACCTTACCAGGTCTTGAC
ATCCTCTGACAACCCTAGAGATAGGGCTTTCCCTTCGGGGACAGAGTGACAGGTGGTGCATGGTTGTCGTCAGCTC
GTGTCGTGAGATGTTGGGTTAAGTCCCGCAACGAGCGCAACCCTTGATCTTAGTTGCCAGCATTCAGTTGGGCACT
CTAAGGTGACTGCCGGTGACAAACCGGAGGAAGGTGGGGATGACGTCAAATCATCATGCCCCTTATGACCTGGGC
TACACACGTGCTACAATGGACAGAACAAAGGGCTGCAAGACCGCAAGGTTTAGCCAATCCCATAAATCTGTTCTC
AGTTCGGATCGCAGTCTGCAACTCGACTGCGTGAAGCTGGAATCGCTAGTAATCGCGGATCAGCATGCCGCGGTG
AATACGTTCCCGGGCCTTGTACACACCGCCCGTCACACCACGAGAGTTTGCAACACCCGAAGTCGGTGAGGTAAC
CTTTATGGAGCCAGCCGCCGAAGGTGGGGCAGATGATTGGGGTGAAG

**Sequences producing significant alignments** Download Select columns Show 100

select all *100 sequences selected* GenBank Graphics Distance tree of results MSA Viewer

| | Description | Scientific Name | Max Score | Total Score | Query Cover | E value | Per. Ident | Acc. Len | Accession |
|---|---|---|---|---|---|---|---|---|---|
| ☑ | Bacillus safensis strain NBRC 100820 16S ribosomal RNA, partial sequence | Bacillus safensis | 2723 | 2723 | 100% | 0.0 | 100.00% | 1474 | NR_113945.1 |
| ☑ | Bacillus australimaris strain MCCC 1A05787 16S ribosomal RNA, partial sequence | Bacillus australimaris | 2712 | 2712 | 100% | 0.0 | 99.86% | 1513 | NR_148787.1 |
| ☑ | Bacillus pumilus strain NBRC 12092 16S ribosomal RNA, partial sequence | Bacillus pumilus | 2712 | 2712 | 100% | 0.0 | 99.86% | 1474 | NR_112637.1 |
| ☑ | Bacillus zhangzhouensis strain MCCC 1A08372 16S ribosomal RNA, partial sequence | Bacillus zhangzhouensis | 2706 | 2706 | 100% | 0.0 | 99.80% | 1513 | NR_148786.1 |
| ☑ | Bacillus altitudinis 41KF2b 16S ribosomal RNA, partial sequence | Bacillus altitudinis 41KF2b | 2689 | 2689 | 100% | 0.0 | 99.59% | 1506 | NR_042337.1 |
