# Supplementary material for: Biochemical and Thermodynamic Studies on a Novel Thermotolerant GH10 Xylanase from Bacillus safensis
Source: Biomolecules. 2022 Jun 6;12(6):790. doi: 10.3390/biom12060790 (PMC9221164; doi:10.3390/biom12060790)
Supplement: Supplementary file 1 [file biomolecules-12-00790-s001.zip › supplementary Figure S3.pdf]

Supplementary Figure S3

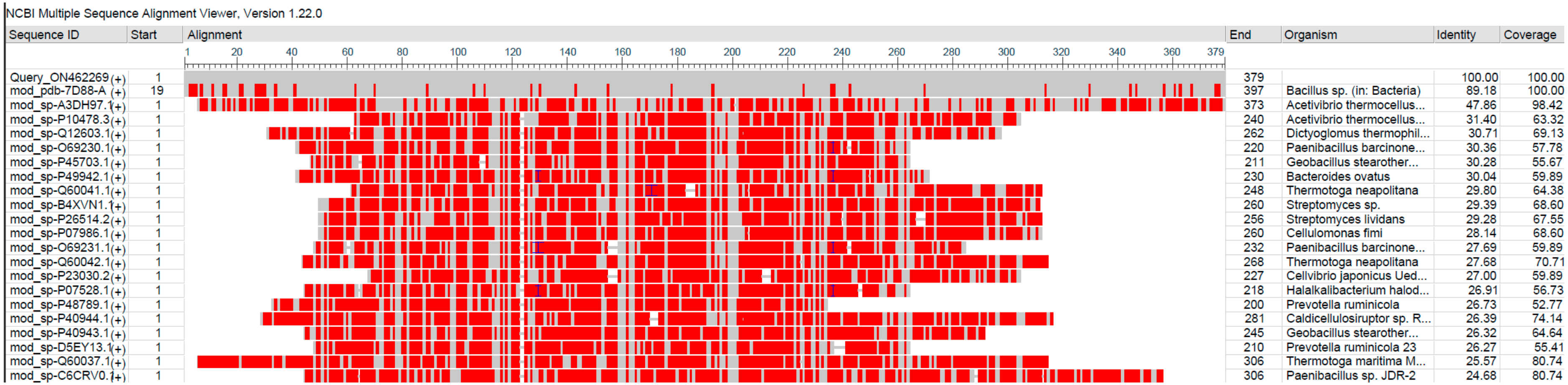

Aminoacid sequence alignment of BsXyn10 with other bacterial GH10 endo-xylanases, using NCBI Multiple Sequence Alignment Viewer Version 1.22.0. Used sequences were obtained from PDB and UniprotKB/Swiss-Prot databases based on maximum sequence identity with BsXyn10. Identical aminoacids are depicted in grey bars.
