# Supplementary material for: Biochemical and Thermodynamic Studies on a Novel Thermotolerant GH10 Xylanase from Bacillus safensis
Source: Biomolecules. 2022 Jun 6;12(6):790. doi: 10.3390/biom12060790 (PMC9221164; doi:10.3390/biom12060790)
Supplement: Supplementary file 1 [file biomolecules-12-00790-s001.zip › supplementary Figure S4.pdf]

Supplementary Figure S4

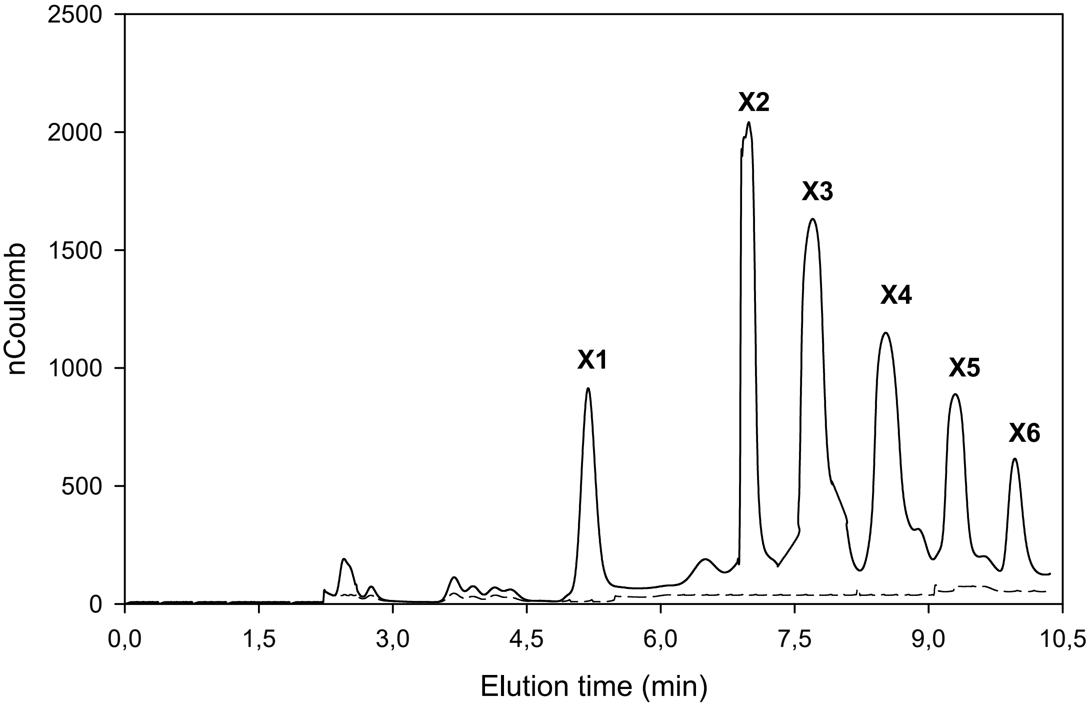

HPAEC-PAD analysis of oat spelt xylan hydrolysis products by BsXyn10 performed as described in Materials and Methods. Dashed line represents the blanks (no addition of BsXyn10). Solid line represents the hydrolysis products after 24 h. Xylose (X1), xylobiose (X2), xylotriose (X3), xylotetraose (X4), xylopentaose (X5) and xylohexaose (X6).
