# Supplementary figures and images for: Biochemical and Thermodynamic Studies on a Novel Thermotolerant GH10 Xylanase from Bacillus safensis
Source: Biomolecules. 2022 Jun 6;12(6):790. doi: 10.3390/biom12060790 (PMC9221164; doi:10.3390/biom12060790)

Supplementary Figure S2

Maps of the plasmids used in the present work.

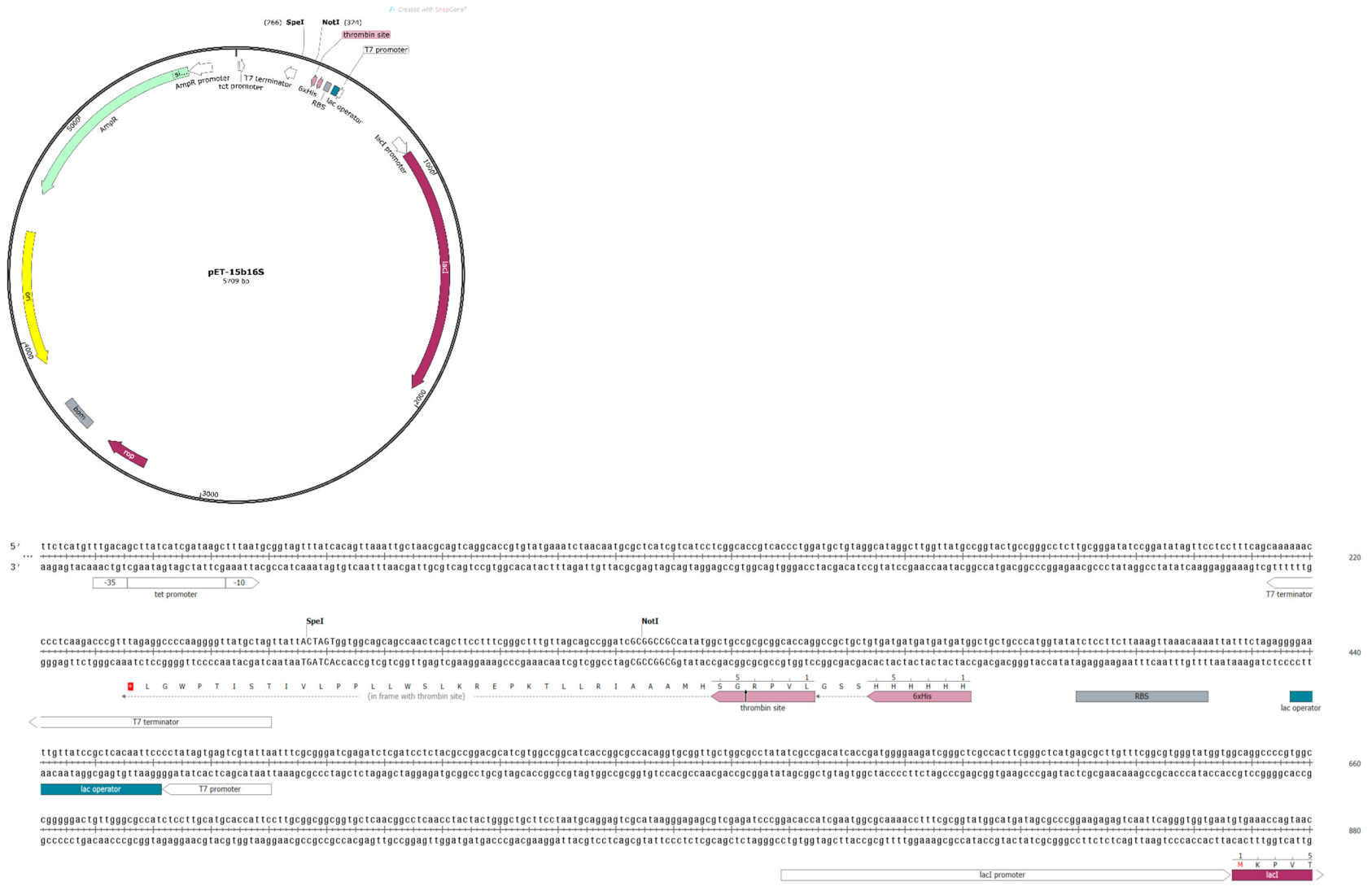

Supplement: Supplementary file 1 [file biomolecules-12-00790-s001.zip › supplementary Figure S2.pdf]
